# Supplementary material for: US and EU Free Trade Agreements and implementation of policies to control tobacco, alcohol, and unhealthy food and drinks: A quasi-experimental analysis
Source: PLoS Med. 2023 Jan 5;20(1):e1004147. doi: 10.1371/journal.pmed.1004147 (PMC9815641; doi:10.1371/journal.pmed.1004147)
Supplement: S1 Supporting Information — (DOCX) [file pmed.1004147.s001.docx]

## Supplementary Appendix

[Appendix A. Matching estimation and covariate balance tests 2](#_Toc121158280)

[Appendix B. Changes from pre-registered protocol 4](#_Toc121158281)

[Table A. Variable measures, data sources and rationale for covariate inclusion 5](#_Toc121158282)

[Table B. List of top 10 largest tobacco, alcohol, and food and drink companies in 2014, 2016, and 2019 by market share and country headquarters 8](#_Toc121158283)

[Table C. Matching model comparison 10](#_Toc121158284)

[Table D. Average Marginal Effect (AME) of US FTA participation on the probability of achieving partial or full implementation and full implementation of unhealthy commodity regulations: logistic regression modelling results 11](#_Toc121158285)

[Table E. Average Marginal Effect (AME) of EU FTA participation on the probability of achieving partial or full implementation and full implementation of unhealthy commodity regulations: logistic regression modelling results 12](#_Toc121158286)

[Table F. Average Marginal Effect of US/EU, US, and EU FTA participation on partial or full and full implementation of risk factor surveys and time-bound NCD targets 13](#_Toc121158287)

[Table G. Average Marginal Effect of participation in FTAs with countries where large tobacco, processed food & drink, and alcohol companies are headquartered on the probability of achieving partial/ full and full implementation of unhealthy commodity regulations 14](#_Toc121158288)

[Table H. Average Marginal Effect of US BITs participation on the implementation of unhealthy commodity regulations after matching 15](#_Toc121158289)

[Table I. Average Marginal Effect of EU BITs participation on the implementation of unhealthy commodity regulations after matching 16](#_Toc121158290)

[Table J. Average Marginal Effect of US FTA participation on partial or full and full implementation of unhealthy commodity regulations after adjusting for participation in US BITS 17](#_Toc121158291)

[Table K. Average Marginal Effect of EU FTA participation on partial or full and full implementation of unhealthy commodity regulations after adjusting for participation in EU BITS 18](#_Toc121158292)

[Table L. Average Marginal Effect of US FTA participation on partial or full of unhealthy commodity regulations: additional robustness checks 19](#_Toc121158293)

[Table M. Average Marginal Effect of EU FTA participation on partial or full of unhealthy commodity regulations: additional robustness checks 20](#_Toc121158294)

### Appendix A. Matching estimation and covariate balance tests

There are substantial differences in the characteristics of countries with and without US/EU FTAs, and it is important to account for these differences when estimating the relationship between US or EU FTA participation and the implementation of unhealthy commodity policies. Randomized assignment into FTAs is not feasible, and so quasi-experimental evaluations are the best feasible approach for assessing potential FTA impacts [1]. Quasi-experimental research designs comprise a variety of approaches designed to support causal inference by re-constructing counterfactual ‘control’ units using available observational data. The choice of approach is often constrained by data availability and the number of available time periods, and matching is suitable when there are few years of data available, as in our study[2,3].

Matching pre-processes the data to identify the ‘untreated’ comparison country or countries without US or EU FTAs that is/are most similar to each country with a EU or US FTAs, thereby reducing imbalance in covariates [2,3]. After the matching, the number and quality of matches can be analysed using a range of diagnostic tests. The matched sample is then analysed using regression models with controls for any remaining covariate imbalances.

We estimated a range of matching models since there are many available indicators of measuring the similarity between treated and untreated countries and several algorithms for placing countries into sub-sets of ‘matches’. For example, a widely used measure of similarity between units is the propensity score, the predicted probability of US or EU FTA participation. However, propensity score matching has been widely criticised, and an alternative measure is the Mahalanobis distance, which is a weighted-measure of similarity in covariates in treated and untreated units [4]. We used both measures and further set a variety of calipers setting the maximum differences on select variables and distance metrics between treated and untreated countries (e.g. maximum difference in GDP per capita of 10,000, maximum difference in the propensity score of 0.05, 0.01, and 0.1 etc). We further deployed a range of different algorithms – nearest neighbour matching, full matching, and covariate-balancing propensity score matching – and set the algorithm to identify the 1- and 3-, and 5- most similar untreated countries, except for full matching which does not impose limits on the number of matches. For all models, we perform exact matches on year and WTO membership status.

After estimating each matching model, we calculated a range of diagnostic tests. We then compare these diagnostic tests across all models and use the best performing model to estimate our final results. Following recommendations in Stuart (2010) and King and Zeng (2006), our covariate balance tests are as follows:

1. The Average Absolute Standardized Difference in characteristics (values closer to 0 indicate greater similarity in matched treated and un-treated groups)
2. The ratio of variance in matched treated and untreated groups (values closer to 1 indicate greater similarity);
3. The complement of the amount of overlap in the covariate distributions between two groups (values closer to 0 indicate grater overlap),
4. Kolmogorov-Smirnov statistics, which measure the greatest distance between the empirical cumulative distribution functions for each variable in the matched treated and untreated groups (values closer to 0 indicting more similar distributions) [3,5].

For simplicity, we sought to use the same matching algorithm for both US and EU FTAs, and so we calculate the above balance statistics for models identifying matched sets for both US and EU FTA participation and take the average. For each FTA participation indicator, we further estimate 3 models with controls for participation in FTAs where large tobacco or alcohol or unhealthy food/ drink companies are headquartered, as in our later models we incorporate different controls for these FTAs depending on the outcome in question (e.g. for tobacco outcomes we control for participation in other FTAs where large tobacco companies are headquartered). The final average of the balance statistics is thus calculated by taking the average of the balance statistics across all 3 sub-categories of models and both US and EU FTAs (n=6 categories of models in total).

Appendix 4 compares the performance of the different matching models we identified. As shown in Appendix 4, the best performing model uses full matching on the Mahalanobis distance with a caliper for GDP within 10,000 USD among matched subsets. Full matching divides the full sample of all treated and all comparison individuals into a series of matched sets. Matching is performed so that the sum of the overall distance metrics across all matched subsets is minimized [6]. Rather than imposing a specific limit on the number of comparison/ treated countries in each set (as with e.g. 1:3 nearest neighbour matching), the sets each contain either 1 treated country and multiple comparison countries, or 1 comparison country and multiple treated countries. The number of treated and comparison countries in each matched set will depend on the relative number of treated and comparison countries with similar Mahalanobis distances, GDP per capita, year, and WTO membership status.

### Appendix B. Changes from pre-registered protocol

All analyses were performed according to our pre-registered protocol, with the following exceptions [7]. First, we did not estimate first-difference models assessing changes over time in the outcome variable as there was an insufficient number of countries with and without US or EU FTAs who changed policy implementation scores, making this analysis insufficiently powered. Second, we were unable to perform heterogeneity analyses assessing variation in the relationship between US/EU FTA participation and policy implementation according to income levels and state capacity. Again, there were insufficient numbers of countries in each sub-group to perform sufficiently powered sub-group analyses. Finally, we did not perform multiple imputation as we were able to identify a sufficient number of matched pairs for our analysis.

### Table A. Variable measures, data sources and rationale for covariate inclusion

| Variable | Measure, range, and data source | Rationale for inclusion |
| --- | --- | --- |
| US FTA participation, EU FTA participation | Indicator of participation in an FTA with the US; indicator of participation in an FTA with the EU.    Binary (0 or 1), from the Design of Trade Agreements Database | Main explanatory variables of interest  References: Dur & Baccini (2014) |
| Democratization | Varieties of Democracy (V-Dem) Multiplicative polyarchy index, which combines scores across multiple indicators of democracy (suffrage, free and fair elections, elected officials, freedom of civil and political organization, and freedom of expression).  Continuous (0-1), from V-Dem Institute, University of Gothenberg | Democracies tend to have more liberal trade policies, and pairs of democracies are more likely to form FTAs. Democratization is also associated with higher NCD implementation scores.  References: Mansfield and Milner (2012); Allen et al. (2020) |
| GDP per capita | GDP per capita, PPP (constant 2017 international $).  Continuous (731 to 155,201), from World Bank World Development Indicators | Countries with and without US/EU FTAs have different levels of GDP per capita, and countries with higher GDP per capita have more resources to implement NCD policies.  References: Egger, Egger & Greenaway (2008); Baier and Bergstrand (2009), Allen et al. (2020) |
| Human capital | The ratio of total enrolment in secondary education (among persons of any age) to the population of the age group that is typically enrolled in secondary education.  Continuous (11.01 to 164), from World Bank World Development Indicators | Differences in skilled labour (which is widespread in the US/EU) affect whether two countries ratify FTAs, whilst the school enrolment rate is a dimension of human capital that is associated with NCD policy implementation  References: Egger, Egger & Greenaway (2008); Allen (2020) |
| Domestic market liberalisation and business regulations | The extent to which the regulatory and infrastructure environments constrain the efficient operation of businesses (excluding trade policies), based on a weighted sum of 13 indicators in the World Bank’s Doing Business report.  Continuous (0-100), from The Heritage Foundation | Trade liberalisation is associated with other reforms to liberalise domestic markets, including limited business regulation, whilst support for minimal business regulation and domestic market liberalisation associates with opposition to, and limited implementation of, unhealthy commodity regulations  References: Billmeier and Nannicini (2013), Rodriguez and Rodrik (2001), Cullerton et al. (2016) |
| WTO Participation | WTO membership status  Binary, from Head et al. (2020) and accessed via Centre d'Etudes Prospectives et d'Informations | WTO participation may incentivise RTA negotiation as governments seek to further liberalise trade (beyond what is possible within WTO), whilst WTO membership may independently influence NCD prevention  References: Baldwin 2009, Barlow et al. 2018 |
| Participation in FTAs with other unhealthy commodity exporters | Indicator of participation in an FTA with countries where the 10 largest tobacco, alcohol, and unhealthy food and drink producers are headquartered, measured according to total sales and market share. The specific control differs according to the outcome being analysed:  Tobacco policies - control for FTAs with countries where the top 10 tobacco companies are headquartered  Alcohol policies - control for FTAs with countries where the top 10 alcohol companies are headquartered  Food and drink policies - control for FTAs with countries where the top 10 food and drink companies are headquartered  Binary (0 or 1)  Sales and market share data from Euromonitor Global Passport.  See S2 for additional detail. | Participation in US/EU FTAs associates with FTA ratification with other unhealthy commodity exporters (as, for example, the US/EU may want to secure the same market advantages as other states), whilst other unhealthy commodity exporters have been found to frequently challenge regulations targeting unhealthy foods, soft-drinks, tobacco and alcohol.  References: Barlow et al. 2018; Dur & Baccini 2014 |
| International political integration | KOF Political Globalisation index, calculated using the number of foreign embassies in a country, personnel contributed to UN security council missions (%),the number of internationally oriented nongovernmental organisations (NGO) operating in that country, the number of multilateral treaties signed since 1945, the number of memberships in international organizations and a measure for the treaty partner diversity.  Continuous, 0-100.  From KOF Swiss Economic Institute | International political integration associates with exchange of information, norms and political pressure regarding both trade policy and NCD prevention, potentially leading governments to seek and negotiate FTAs whilst also influencing policies targeting unhealthy commodities.  References: Dobbin et al. 2007; Valente et al. 2019 |
| International economic integration | KOF de facto Economic Globalisation index, calculated using total trade in goods and services, trade diversity, FDI and portfolio investment, debt, reserves, and international income payments.  Continuous, 0-100.  From KOF Swiss Economic Institute. | Countries with US/EU FTAs also tend to have larger trade and investment volumes (independent of the effects of US/EU FTAs) as, for example, the importance of trade to the national economy can lead countries to pursue FTAs, and countries may limit/ be pressured to limit policies targeting unhealthy commodities in an attempt to minimize trade disruptions.  References: Baier & Bergstrand (2009); Barlow et al. 2018 |

Notes: a: all variables are lagged by one year, with the exception of secondary education for which we lag the variable by 2 years in 2020 due to limited data availability in 2019. Table shows data availability 2014, 2016 and 2019. Ranges show min to max in this period if not equal across years. See bibliography for full references [8–18].

### Table B. List of top 10 largest tobacco, alcohol, and food and drink companies in 2014, 2016, and 2019 by market share and country headquarters

| Product category | Company | Headquarters | Years in top 10 |
| --- | --- | --- | --- |
| *Tobacco* |  |  |  |
|  | China Nat Tobacco Corp | China | 2014, 2016, 2019 |
|  | British American Tobacco | UK | 2014, 2016, 2019 |
|  | Philip Morris | USA | 2014, 2016, 2019 |
|  | Japan Tobacco | Japan | 2014, 2016, 2019 |
|  | Imperial Brands | UK | 2014, 2016, 2019 |
|  | Altria Group Inc | USA | 2014, 2016, 2019 |
|  | Reynolds | USA | 2014, 2016 |
|  | KT&G | South Korea | 2014, 2016, 2019 |
|  | Gudang Garam | Indonesia | 2014, 2016, 2019 |
|  | ITC | India | 2014, 2019 |
|  | Eastern Co SAE | Egypt | 2016, 2019 |
| *Alcohol* |  |  |  |
|  | AnnBu InBev | Belgium | 2014, 2016, 2019 |
|  | Heineken | Netherlands | 2014, 2016, 2019 |
|  | Carlsberg | Denmark | 2014, 2016, 2019 |
|  | China Resources Holdings Co | China | 2014, 2016, 2019 |
|  | Molson Coors | USA | 2014, 2016, 2019 |
|  | Tsingtao | Japan | 2014, 2016, 2019 |
|  | Asahi | Japan | 2016, 2019 |
|  | Diageo | UK | 2014, 2016, 2019 |
|  | Beijing Yanjing | China | 2014, 2016, 2019 |
|  | SABMiller Ltd | UK | 2014 |
|  | Kirin Holdings Co Ltd | Japan | 2014, 2016 |
|  | Thai Beverage TCL | Thailand | 2019 |
| *Food and soft-drinks* |  |  |  |
| Carbonated soft-drinks |  |  |  |
|  | Coca-Cola Co, The | USA | 2014, 2016, 2019 |
|  | PepsiCo Inc | USA | 2014, 2016, 2019 |
|  | Dr Pepper Snapple Group Inc^a^ | USA | 2014, 2016, 2019 |
|  | Aje Group | Peru | 2014, 2016, 2019 |
|  | Anheuser-Busch InBev NV | Belgium | 2014, 2016, 2019 |
|  | Suntory Holdings Ltd | Japan | 2014, 2016, 2019 |
|  | Asahi Group Holdings Ltd | Japan | 2014, 2016, 2019 |
|  | Consorcio Aga SA de CV | Mexico | 2014, 2016,2019 |
|  | Kirin Holdings Co Ltd | Japan | 2014, 2016 |
|  | Postobón SA | Colombia | 2014, 2016, 2019 |
|  | Refres Now SA | Argentina | 2019 |
| Pre-packaged meals and cooking ingredients |  |  |  |
|  | Nestlé SA | Switzerland | 2014, 2016, 2019 |
|  | Unilever Group | UK | 2014, 2016, 2019 |
|  | Wilmar International Ltd | Singapore | 2014, 2016, 2019 |
|  | Heinz Co, HJ^b^ | USA | 2014 |
|  | Kraft Foods Group Inc^b^ | USA | 2014 |
|  | Kraft Heinz Co | USA | 2016, 2019 |
|  | Campbell Soup Co | USA | 2014, 2016, 2019 |
|  | ConAgra Foods Inc | USA | 2014, 2016, 2019 |
|  | McCormick & Co Inc | USA | 2014, 2016, 2019 |
|  | General Mills Inc | USA | 2014, 2016, 2019 |
|  | Ajinomoto Co Inc | Japan | 2014, 2016, 2019 |
|  | Ferrero & related parties | Italy | 2016 |
|  | Adani Group | India | 2019 |
| Pre-packaged snacks |  |  |  |
|  | Mondelez International Inc | USA | 2014, 2016, 2019 |
|  | PepsiCo Inc | USA | 2014, 2016, 2019 |
|  | Mars Inc | USA | 2014, 2016, 2019 |
|  | Nestlé SA | Switzerland | 2014, 2016, 2019 |
|  | Unilever Group | UK | 2014, 2016, 2019 |
|  | Ferrero & related parties | Italy | 2014, 2016, 2019 |
|  | Hershey Co, The | USA | 2014, 2016, 2019 |
|  | Kellogg Co | USA | 2014, 2016, 2019 |
|  | General Mills Inc | USA | 2014, 2016, 2019 |
|  | Chocoladefabriken Lindt & Sprüngli AG | Switzerland | 2014, 2016, 2019 |
| Baby food |  |  |  |
|  | Nestlé SA | Switzerland | 2014, 2016, 2019 |
|  | Danone, Groupe | France | 2014, 2016, 2019 |
|  | Mead Johnson Nutrition Co | USA | 2014, 2016, 2019 |
|  | Abbott Laboratories Inc | USA | 2014, 2016, 2019 |
|  | Royal FrieslandCampina NV | Netherlands | 2014, 2016, 2019 |
|  | Heinz Co, HJb | USA | 2014, 2016, 2019 |
|  | Hangzhou Beingmate Group Co Ltd | China | 2014 |
|  | Inner Mongolia Yili Industrial Group Co Ltd | China | 2014, 2016, 2019 |
|  | Feihe International Inc | China | 2014, 2016, 2019 |
|  | Hipp GmbH & Co Vertrieb KG | Switzerland | 2014, 2016 |
|  | Biostime International Holdings Ltd | China | 2016 |
|  | Reckitt Benckiser Group Plc (RB) | UK | 2019 |
|  | Shijiazhuang Junlebao Milk Co Ltd | China | 2019 |
|  | Australia Ausnutria Dairy Pty Ltd | Australia | 2019 |

Notes: We control for participation in FTAs where the companies above were headquartered if they ranked in the top 10 in all 3 years. We incorporate separate controls for tobacco, alcohol, and food/drink-related policy outcomes, with respective controls for participation in FTAs where major tobacco, alcohol, and pre-packaged food and drink companies were headquartered. List compiled using Euromonitor Global Passport. Market share calculated as a percentage of total recorded sales. Data pertain to top 10 companies by market share. There are few changes in this ranking through to 2020. a: Keurig Dr Pepper Inc in 2019; b: Merged to Kraft Heinz Co in 2016 and 2019.

### Table C. Matching model comparison

| Model | Mean n | Mean AASD^a^ | Variance Ratio^b^ | Overlapping Coefficient^c^ | KS Statistic^d^ |
| --- | --- | --- | --- | --- | --- |
| Full matching on Mahalanobis distance, caliper for GDP within 10,000 USD^f^ | 44 | 0.04 | 0.81 | 0.12 | 0.14 |
| Full matching on propensity score, caliper for GDP within 10,000 USD^f^ | 44 | 0.08 | 0.92 | 0.14 | 0.16 |
| Full matching on propensity score, caliper for GDP within 7,500 USD^f^ | 43 | 0.05 | 0.79 | 0.12 | 0.13 |
| Full matching on Mahalanobis distance, caliper for GDP within 7,500 USD | 43 | 0.05 | 0.82 | 0.12 | 0.13 |
| Full matching on Mahalanobis distance, caliper for GDP within 7,500 USD | 43 | 0.07 | 0.95 | 0.15 | 0.17 |
| Full matching on Mahalanobis distance, caliper for GDP within 5,000 USD | 43 | 0.08 | 0.9 | 0.14 | 0.16 |
| 1:1 nearest neighbour, Mahalanobis distance | 25 | 0.06 | 1.05 | 0.06 | 0.09 |
| 1:3 nearest neighbours, Mahalanobis distance | 25 | 0.08 | 1.07 | 0.07 | 0.09 |
| 1:5 nearest neighbours, Mahalanobis distance | 25 | 0.08 | 1.04 | 0.07 | 0.09 |
| 1:1 nearest neighbour, Mahalanobis distance | 11 | 0.03 | 1.22 | 0.09 | 0.13 |
| 1:3 nearest neighbours, propensity score, with a caliper of 0.1 | 6 | 0.03 | 1.69 | 0.08 | 0.12 |
| 1:5 nearest neighbours, propensity score, with a caliper of 0.1 | 6 | 0.04 | 1.69 | 0.08 | 0.12 |
| 1:3 nearest neighbours, covariate-balancing propensity score, with a caliper of 0.1 | 6 | 0.06 | 1.95 | 0.08 | 0.13 |
| 1:5 nearest neighbours, covariate-balancing propensity score, with a caliper of 0.1 | 6 | 0.06 | 1.97 | 0.08 | 0.13 |
| 1:1 nearest neighbours, covariate-balancing propensity score, with a caliper of 0.1 | 6 | 0.08 | 1.93 | 0.09 | 0.13 |

Notes: Mean sample sizes and balance statistics are calculated by taking the mean across the 3-sub categories of models identifying matched sets for US FTA and EU participation (n=6 in total). a – Average Absolute Standardized Difference; c – Ratio of variance in matched treated and untreated group; c – the complement of the amount of overlap in the covariate distributions between two groups; d – Kolmogorov-Smirnov statistics. See Appendix 3 for further explanation.

### Table D. Average Marginal Effect (AME) of US FTA participation on the probability of achieving partial or full implementation and full implementation of unhealthy commodity regulations: logistic regression modelling results

|  | Partial or full implementation | | | | Full implementation | | | | |
| --- | --- | --- | --- | --- | --- | --- | --- | --- | --- |
|  | **No controls** | | **Full controls^b^** | | **No controls** | | | **Full controls** | |
| Regulation/ policy | **AME^a^ (SE)** | **95% CI** | **AME (SE)** | **95% CI** | **AME (SE)** | **95% CI** | **AME (SE)** | | **95% CI** |
| Tobacco taxes | -0.02 (0.07) | -0.16 to 0.13 | 0.07 (0.07) | -0.06 to 0.20 | -0.06 (0.05) | -0.16 to 0.04 | 0.00 (0.06) | | -0.12 to 0.13 |
| Smoke free places | 0.07 (0.07) | -0.06 to 0.20 | -0.09 (0.10) | -0.29 to 0.10 | 0.22 (0.08) | 0.07 to 0.37 | -0.08 (0.07) | | -0.22 to 0.07 |
| Graphic warnings | -0.01 (0.05) | -0.12 to 0.10 | -0.14 (0.07) | -0.28 to 0.00 | -0.04 (0.08) | -0.19 to 0.11 | -0.26 (0.07) | | -0.4 to -0.13 |
| Tobacco ad bans | -0.24 (0.07) | -0.38 to -0.09 | -0.15 (0.08) | -0.31 to 0.02 | -0.01 (0.06) | -0.13 to 0.11 | -0.05 (0.07) | | -0.18 to 0.08 |
| Alcohol ad/ sales restrictions | -0.13 (0.07) | -0.26 to 0.00 | -0.02 (0.10) | -0.22 to 0.17 | -0.02 (0.04) | -0.11 to 0.06 | 0.00 (0.04) | | -0.08 to 0.08 |
| Alcohol taxes | 0.05 (0.06) | -0.07 to 0.17 | 0.01 (0.09) | -0.17 to 0.18 | 0.07 (0.07) | -0.07 to 0.21 | 0.01 (0.08) | | -0.15 to 0.17 |
| Salt reduction | 0.00 (0.07) | -0.14 to 0.15 | 0.02 (0.07) | -0.12 to 0.17 | -0.04 (0.07) | -0.17 to 0.09 | -0.03 (0.08) | | -0.18 to 0.12 |
| Fat limits and bans | -0.02 (0.07) | -0.16 to 0.13 | 0.04 (0.07) | -0.11 to 0.18 | -0.05 (0.07) | -0.19 to 0.09 | 0.04 (0.07) | | -0.11 to 0.18 |
| Child marketing restrictions | -0.02 (0.07) | -0.16 to 0.13 | 0.11 (0.08) | -0.04 to 0.27 | -0.02 (0.07) | -0.16 to 0.13 | 0.11 (0.08) | | -0.04 to 0.27 |
| Breast milk code | 0.11 (0.08) | -0.05 to 0.27 | 0.16 (0.08) | -0.01 to 0.32 | 0.19 (0.08) | 0.03 to 0.35 | 0.14 (0.09) | | -0.04 to 0.32 |

Notes: a - AME: Average Marginal Effect (difference in predicted probability of achieving partial or full implementation, and full implementation, with and without US FTAs). b – Models control for GDP per capita, democratization, the proportion of the eligible population with secondary education, WTO participation, international political integration, and participation in an FTA with other countries where large tobacco, food or alcohol companies are headquartered.

### Table E. Average Marginal Effect (AME) of EU FTA participation on the probability of achieving partial or full implementation and full implementation of unhealthy commodity regulations: logistic regression modelling results

|  | Partial or full implementation | | | | Full implementation | | | | |
| --- | --- | --- | --- | --- | --- | --- | --- | --- | --- |
|  | **No controls** | | **Full controls^a^** | | **No controls** | | | **Full controls** | |
| Regulation/ policy | **AME^b^ (SE)** | **95% CI** | **AME (SE)** | **95% CI** | **AME (SE)** | **95% CI** | **AME (SE)** | | **95% CI** |
| Tobacco taxes | -0.21 (0.07) | -0.36 to -0.07 | -0.10 (0.07) | -0.24 to 0.05 | -0.08 (0.05) | -0.19 to 0.02 | -0.05 (0.07) | | -0.17 to 0.08 |
| Smoke free places | 0.23 (0.05) | 0.12 to 0.33 | -0.02 (0.13) | -0.28 to 0.24 | 0.53 (0.07) | 0.40 to 0.66 | 0.27 (0.12) | | 0.04 to 0.50 |
| Graphic warnings | -0.09 (0.07) | -0.22 to 0.04 | -0.21 (0.07) | -0.34 to -0.08 | 0.08 (0.08) | -0.08 to 0.24 | -0.27 (0.08) | | -0.43 to -0.10 |
| Tobacco ad bans | -0.24 (0.08) | -0.39 to -0.09 | -0.08 (0.10) | -0.27 to 0.10 | 0.09 (0.07) | -0.05 to 0.22 | 0.04 (0.08) | | -0.12 to 0.21 |
| Alcohol ad/ sales restrictions | -0.29 (0.05) | -0.39 to -0.18 | -0.24 (0.08) | -0.39 to -0.08 | 0.04 (0.01) | 0.02 to 0.07 | 0.04 (0.01) | | 0.02 to 0.07 |
| Alcohol taxes | 0.11 (0.06) | -0.01 to 0.22 | 0.16 (0.07) | 0.03 to 0.29 | 0.20 (0.08) | 0.03 to 0.36 | 0.17 (0.12) | | -0.07 to 0.42 |
| Salt reduction | -0.13 (0.07) | -0.27 to 0.00 | 0.01 (0.08) | -0.15 to 0.16 | -0.14 (0.06) | -0.27 to -0.01 | 0.05 (0.09) | | -0.13 to 0.22 |
| Fat limits and bans | -0.16 (0.07) | -0.3 to -0.02 | -0.03 (0.08) | -0.18 to 0.13 | -0.21 (0.06) | -0.34 to -0.08 | -0.05 (0.08) | | -0.21 to 0.11 |
| Child marketing restrictions | -0.27 (0.06) | -0.38 to -0.15 | -0.24 (0.08) | -0.40 to -0.09 | -0.27 (0.06) | -0.38 to -0.15 | -0.24 (0.08) | | -0.40 to -0.09 |
| Breast milk code | 0.11 (0.08) | -0.05 to 0.27 | 0.05 (0.10) | -0.14 to 0.24 | 0.16 (0.08) | 0.00 to 0.31 | 0.09 (0.10) | | -0.10 to 0.28 |

Notes: a – Models control for GDP per capita, democratization, the proportion of the eligible population with secondary education, WTO participation, international political integration, and participation in an FTA with other countries where large tobacco, food or alcohol companies are headquartered; b - AME: Average Marginal Effect (difference in predicted probability of achieving partial or full implementation, and full implementation, with and without EU FTAs).

### Table F. Average Marginal Effect of US/EU, US, and EU FTA participation on partial or full and full implementation of risk factor surveys and time-bound NCD targets

1. Risk factor surveys

|  |  | |  | |  | |  |
| --- | --- | --- | --- | --- | --- | --- | --- |
|  | **Partial or full implementation** | | | | **Full implementation** | | |
| FTA indicator | **Beta (SE)** | **95% CI** | | **Beta (SE)** | | **95% CI** | |
| US FTA | 0.10 (0.03) | 0.05 to 0.16 | | 0.02 (0.07) | | -0.12 to 0.17 | |
| EU FTA | 0.00 (0.12) | -0.25 to 0.24 | | 0.17 (0.12) | | -0.08 to 0.41 | |

1. Time-bound NCD targets

|  |  | |  | |  | |  |
| --- | --- | --- | --- | --- | --- | --- | --- |
|  | **Partial or full implementation** | | | | **Full implementation** | | |
| FTA indicator | **Beta (SE)** | **95% CI** | | **Beta (SE)** | | **95% CI** | |
| US FTA | -0.20 (0.12) | -0.44 to 0.04 | | -0.14 (0.12) | | -0.37 to 0.08 | |
| EU FTA | 0.10 (0.13) | -0.15 to 0.34 | | 0.37 (0.12) | | 0.14 to 0.60 | |

### Table G. Average Marginal Effect of participation in FTAs with countries where large tobacco, processed food & drink, and alcohol companies are headquartered on the probability of achieving partial/ full and full implementation of unhealthy commodity regulations


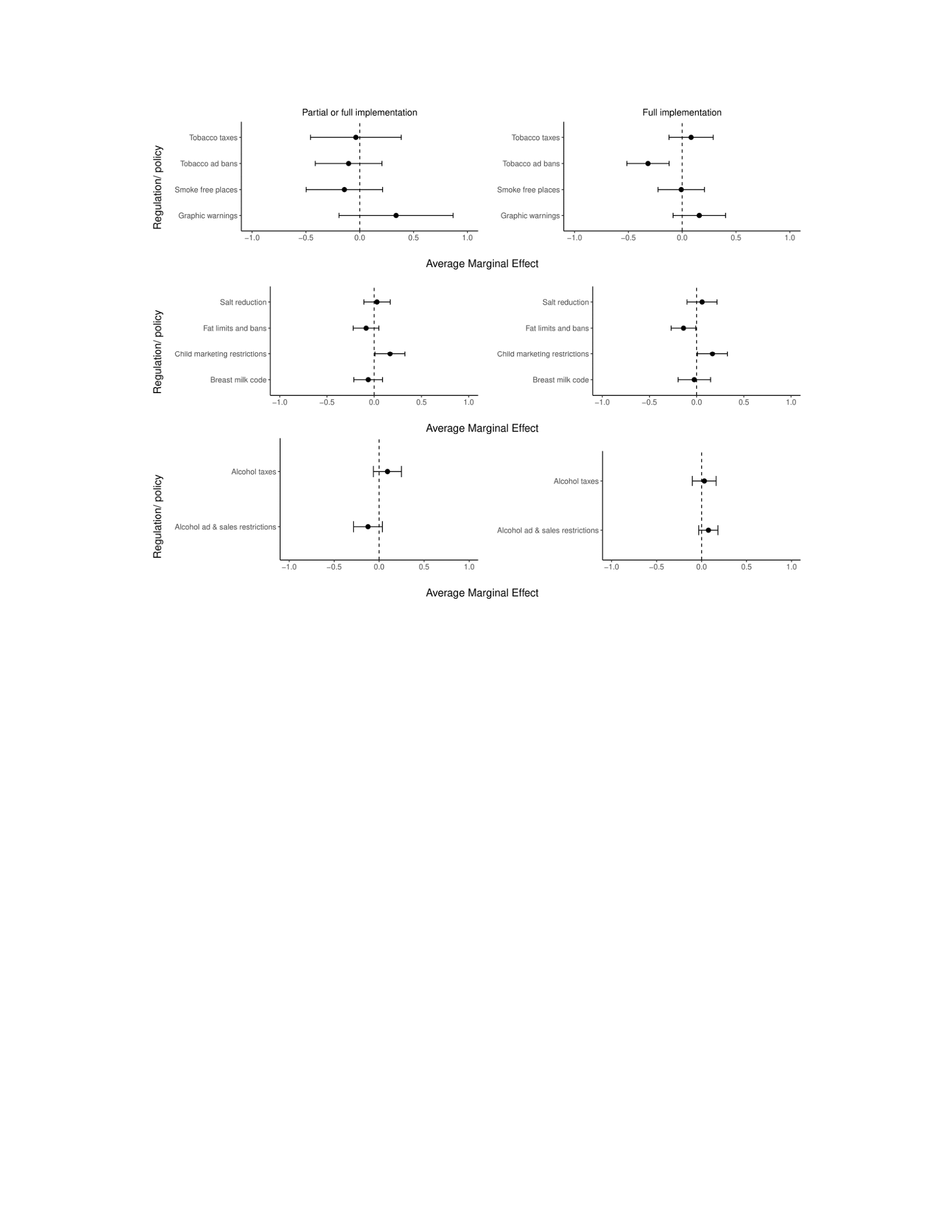


### Table H. Average Marginal Effect of US BITs participation on the implementation of unhealthy commodity regulations after matching

### *Table I. Average Marginal Effect of EU BITs participation on the implementation of unhealthy commodity regulations after matching*

### Table J. Average Marginal Effect of US FTA participation on partial or full and full implementation of unhealthy commodity regulations after adjusting for participation in US BITS

|  |  |  |  |  |  |  |  |  |
| --- | --- | --- | --- | --- | --- | --- | --- | --- |
|  | **Partial or full implementation** | | | | **Full implementation** | | | |
|  | **Original model** | | **With US BIT control** | | **Original model** | | **With US BIT control** | |
|  | **Beta (SE)** | **95% CI** | **Beta (SE)** | **95% CI** | **Beta (SE)** | **95% CI** | **Beta (SE)** | **95% CI** |
| Tobacco taxes | 0.05 (0.07) | -0.08 to 0.18 | 0.02 (0.09) | -0.15 to 0.20 | 0.05 (0.13) | -0.20 to 0.30 | -0.02 (0.12) | -0.25 to 0.21 |
| Smoke free places | -0.53 (0.05) | -0.63 to -0.43 | -0.55 (0.05) | -0.65 to -0.45 | -0.24 (0.08) | -0.39 to -0.08 | -0.24 (0.08) | -0.39 to -0.08 |
| Graphic warnings | -0.09 (0.21) | -0.50 to 0.32 | -0.11 (0.20) | -0.51 to 0.29 | -0.37 (0.07) | -0.51 to -0.22 | -0.37 (0.09) | -0.54 to -0.20 |
| Tobacco ad bans | 0.01 (0.09) | -0.16 to 0.18 | 0.02 (0.09) | -0.15 to 0.19 | 0.05 (0.12) | -0.19 to 0.29 | 0.10 (0.12) | -0.13 to 0.34 |
| Alcohol ad & sales restrictions | -0.03 (0.13) | -0.29 to 0.23 | -0.04 (0.14) | -0.31 to 0.23 | 0.03 (0.02) | -0.02 to 0.07 | 0.02 (0.03) | -0.04 to 0.08 |
| Alcohol taxes | -0.01 (0.09) | -0.19 to 0.17 | -0.01 (0.09) | -0.19 to 0.17 | -0.05 (0.09) | -0.23 to 0.14 | -0.02 (0.09) | -0.21 to 0.16 |
| Salt reduction | -0.01 (0.11) | -0.23 to 0.21 | -0.02 (0.10) | -0.22 to 0.17 | -0.04 (0.12) | -0.27 to 0.19 | -0.04 (0.11) | -0.25 to 0.18 |
| Fat limits and bans | -0.04 (0.10) | -0.24 to 0.16 | -0.05 (0.10) | -0.24 to 0.15 | -0.07 (0.10) | -0.26 to 0.12 | -0.05 (0.10) | -0.24 to 0.14 |
| Child marketing restrictions | 0.12 (0.09) | -0.06 to 0.30 | 0.14 (0.09) | -0.03 to 0.31 | 0.12 (0.09) | -0.06 to 0.30 | 0.14 (0.09) | -0.03 to 0.31 |
| Breast milk code | 0.18 (0.11) | -0.03 to 0.40 | 0.19 (0.11) | -0.02 to 0.40 | 0.19 (0.13) | -0.06 to 0.45 | 0.21 (0.13) | -0.04 to 0.47 |

Notes: shading shows statistically significant results.

### Table K. Average Marginal Effect of EU FTA participation on partial or full and full implementation of unhealthy commodity regulations after adjusting for participation in EU BITS

|  |  |  |  |  |  |  |  |  |
| --- | --- | --- | --- | --- | --- | --- | --- | --- |
|  | **Partial or full implementation** | | | | **Full implementation** | | | |
|  | **Original model** | | **With EU member BIT control** | | **Original model** | | **With EU member BIT control** | |
|  | **Beta (SE)** | **95% CI** | **Beta (SE)** | **95% CI** | **Beta (SE)** | **95% CI** | **Beta (SE)** | **95% CI** |
| Tobacco taxes | -0.06 (0.06) | -0.17 to 0.05 | -0.06 (0.09) | -0.25 to 0.12 | -0.05 (0.10) | -0.25 to 0.14 | -0.10 (0.11) | -0.31 to 0.12 |
| Smoke free places | -0.05 (0.06) | -0.16 to 0.07 | -0.11 (0.09) | -0.29 to 0.07 | 0.14 (0.13) | -0.11 to 0.39 | 0.16 (0.12) | -0.09 to 0.40 |
| Graphic warnings | -0.25 (0.06) | -0.36 to -0.13 | -0.24 (0.05) | -0.34 to -0.14 | -0.28 (0.09) | -0.45 to -0.10 | -0.26 (0.08) | -0.43 to -0.09 |
| Tobacco ad bans | -0.07 (0.12) | -0.31 to 0.16 | -0.05 (0.12) | -0.28 to 0.19 | -0.04 (0.12) | -0.27 to 0.18 | -0.02 (0.10) | -0.22 to 0.18 |
| Alcohol ad & sales restrictions | -0.22 (0.13) | -0.48 to 0.05 | -0.26 (0.13) | -0.51 to 0.00 | -0.30 (0.19) | -0.66 to 0.06 | 0.00 (0.15) | -0.29 to 0.29 |
| Alcohol taxes | 0.01 (0.12) | -0.24 to 0.25 | -0.05 (0.12) | -0.28 to 0.19 | 0.20 (0.10) | -0.01 to 0.40 | 0.20 (0.10) | 0.00 to 0.40 |
| Salt reduction | 0.11 (0.10) | -0.08 to 0.31 | 0.09 (0.11) | -0.13 to 0.31 | 0.14 (0.09) | -0.03 to 0.30 | 0.19 (0.08) | 0.03 to 0.35 |
| Fat limits and bans | 0.16 (0.07) | 0.01 to 0.30 | 0.16 (0.09) | -0.02 to 0.33 | 0.11 (0.09) | -0.06 to 0.28 | 0.13 (0.09) | -0.04 to 0.30 |
| Child marketing restrictions | -0.25 (0.11) | -0.47 to -0.03 | -0.16 (0.11) | -0.39 to 0.06 | -0.25 (0.11) | -0.47 to -0.03 | -0.24 (0.11) | -0.45 to -0.03 |
| Breast milk code | 0.14 (0.10) | -0.07 to 0.34 | 0.13 (0.13) | -0.12 to 0.37 | 0.09 (0.11) | -0.13 to 0.30 | 0.14 (0.11) | -0.08 to 0.36 |

Notes: shading shows statistically significant results.

### Table L. Average Marginal Effect of US FTA participation on partial or full of unhealthy commodity regulations: additional robustness checks

1. **Partial or full implementation**

|  | **Original model** | | **With other imp control** | | **With control for total FTAs** | | **With control for US BITs, EU BITs, EU FTAs, and Total FTAs** | |
| --- | --- | --- | --- | --- | --- | --- | --- | --- |
|  | **Beta (SE)** | **95% CI** | **Beta (SE)** | **95% CI** | **Beta (SE)** | **95% CI** | **Beta (SE)** | **95% CI** |
| Tobacco taxes | 0.05 (0.07) | -0.08 to 0.18 | 0.09 (0.07) | -0.04 to 0.23 | -0.02 (0.06) | -0.15 to 0.11 | -0.03 (0.07) | -0.16 to 0.10 |
| Smoke free places | -0.53 (0.05) | -0.63 to -0.43 | -0.55 (0.05) | -0.65 to -0.45 | -0.54 (0.05) | -0.64 to -0.44 | -0.44 (0.10) | -0.64 to -0.24 |
| Graphic warnings | -0.09 (0.21) | -0.50 to 0.32 | -0.10 (0.21) | -0.51 to 0.32 | -0.19 (0.14) | -0.45 to 0.08 | -0.21 (0.08) | -0.36 to -0.06 |
| Tobacco ad bans | 0.01 (0.09) | -0.16 to 0.18 | 0.01 (0.08) | -0.14 to 0.16 | -0.07 (0.09) | -0.25 to 0.11 | -0.12 (0.12) | -0.36 to 0.11 |
| Alcohol ad & sales restrictions | -0.03 (0.13) | -0.29 to 0.23 | -0.03 (0.13) | -0.28 to 0.22 | -0.04 (0.11) | -0.26 to 0.18 | 0.01 (0.13) | -0.25 to 0.26 |
| Alcohol taxes | -0.01 (0.09) | -0.19 to 0.17 | 0.01 (0.10) | -0.18 to 0.20 | -0.03 (0.10) | -0.23 to 0.17 | -0.02 (0.13) | -0.27 to 0.23 |
| Salt reduction | -0.01 (0.11) | -0.23 to 0.21 | -0.06 (0.10) | -0.26 to 0.15 | -0.01 (0.12) | -0.24 to 0.22 | -0.03 (0.11) | -0.24 to 0.19 |
| Fat limits and bans | -0.04 (0.10) | -0.24 to 0.16 | -0.05 (0.10) | -0.25 to 0.15 | -0.01 (0.11) | -0.22 to 0.20 | -0.03 (0.13) | -0.28 to 0.22 |
| Child marketing restrictions | 0.12 (0.09) | -0.06 to 0.30 | 0.09 (0.10) | -0.09 to 0.28 | 0.10 (0.10) | -0.09 to 0.29 | 0.12 (0.12) | -0.11 to 0.34 |
| Breast milk code | 0.18 (0.11) | -0.03 to 0.40 | 0.13 (0.11) | -0.08 to 0.35 | 0.22 (0.12) | 0.00 to 0.45 | -0.07 (0.13) | -0.34 to 0.19 |

1. **Full implementation**

|  | **Original model** | | **With other imp control** | | **With control for total FTAs** | | **With control for US BITs, EU BITs, EU FTAs, and Total FTAs** | |
| --- | --- | --- | --- | --- | --- | --- | --- | --- |
|  | **Beta (SE)** | **95% CI** | **Beta (SE)** | **95% CI** | **Beta (SE)** | **95% CI** | **Beta (SE)** | **95% CI** |
| Tobacco taxes | 0.05 (0.13) | -0.20 to 0.30 | 0.06 (0.13) | -0.18 to 0.31 | 0.14 (0.12) | -0.10 to 0.38 | -0.09 (0.14) | -0.36 to 0.17 |
| Smoke free places | -0.24 (0.08) | -0.39 to -0.08 | -0.20 (0.08) | -0.36 to -0.04 | -0.20 (0.07) | -0.34 to -0.05 | -0.17 (0.11) | -0.40 to 0.05 |
| Graphic warnings | -0.37 (0.07) | -0.51 to -0.22 | -0.33 (0.09) | -0.51 to -0.16 | -0.34 (0.08) | -0.50 to -0.19 | -0.24 (0.10) | -0.44 to -0.03 |
| Tobacco ad bans | 0.05 (0.12) | -0.19 to 0.29 | 0.02 (0.11) | -0.20 to 0.24 | 0.06 (0.11) | -0.16 to 0.28 | 0.01 (0.15) | -0.28 to 0.29 |
| Alcohol ad & sales restrictions | 0.03 (0.02) | -0.02 to 0.07 | 0.03 (0.04) | -0.05 to 0.11 | 0.08 (0.04) | 0.01 to 0.15 | 0.09 (0.10) | -0.12 to 0.29 |
| Alcohol taxes | -0.05 (0.09) | -0.23 to 0.14 | -0.05 (0.08) | -0.20 to 0.10 | -0.09 (0.08) | -0.24 to 0.07 | 0.12 (0.17) | -0.22 to 0.46 |
| Salt reduction | -0.04 (0.12) | -0.27 to 0.19 | -0.08 (0.11) | -0.29 to 0.13 | -0.03 (0.12) | -0.26 to 0.20 | 0.05 (0.14) | -0.22 to 0.33 |
| Fat limits and bans | -0.07 (0.10) | -0.26 to 0.12 | -0.07 (0.10) | -0.26 to 0.13 | -0.05 (0.09) | -0.24 to 0.13 | 0.09 (0.15) | -0.20 to 0.39 |
| Child marketing restrictions | 0.12 (0.09) | -0.06 to 0.30 | 0.09 (0.10) | -0.09 to 0.28 | 0.10 (0.10) | -0.09 to 0.29 | 0.15 (0.14) | -0.13 to 0.43 |
| Breast milk code | 0.19 (0.13) | -0.06 to 0.45 | 0.13 (0.13) | -0.14 to 0.39 | 0.24 (0.12) | 0.00 to 0.47 | 0.10 (0.18) | -0.24 to 0.45 |

Notes: shading shows statistically significant results.

### Table M. Average Marginal Effect of EU FTA participation on partial or full of unhealthy commodity regulations: additional robustness checks

1. **Partial or full implementation**

|  | **Original model** | | **With other imp control** | | **With control for total FTAs** | | **With control for US BITs, US FTAs, EU BITs, and Total FTAs** | |
| --- | --- | --- | --- | --- | --- | --- | --- | --- |
|  | **Beta (SE)** | **95% CI** | **Beta (SE)** | **95% CI** | **Beta (SE)** | **95% CI** | **Beta (SE)** | **95% CI** |
| Tobacco taxes | -0.06 (0.06) | -0.17 to 0.05 | -0.23 (0.09) | -0.40 to -0.06 | -0.14 (0.08) | -0.30 to 0.02 | -0.25 (0.09) | -0.43 to -0.07 |
| Smoke free places | -0.05 (0.06) | -0.16 to 0.07 | -0.08 (0.06) | -0.20 to 0.04 | -0.07 (0.06) | -0.19 to 0.05 | -0.22 (0.09) | -0.39 to -0.04 |
| Graphic warnings | -0.25 (0.06) | -0.36 to -0.13 | -0.25 (0.06) | -0.37 to -0.12 | -0.25 (0.06) | -0.37 to -0.14 | -0.28 (0.07) | -0.41 to -0.15 |
| Tobacco ad bans | -0.07 (0.12) | -0.31 to 0.16 | -0.12 (0.12) | -0.35 to 0.11 | -0.09 (0.13) | -0.34 to 0.17 | -0.18 (0.12) | -0.40 to 0.05 |
| Alcohol ad & sales restrictions | -0.22 (0.13) | -0.48 to 0.05 | -0.27 (0.13) | -0.52 to -0.02 | -0.27 (0.13) | -0.52 to -0.01 | -0.21 (0.14) | -0.48 to 0.06 |
| Alcohol taxes | 0.01 (0.12) | -0.24 to 0.25 | 0.06 (0.10) | -0.14 to 0.25 | 0.05 (0.11) | -0.17 to 0.26 | 0.04 (0.12) | -0.19 to 0.27 |
| Salt reduction | 0.11 (0.10) | -0.08 to 0.31 | 0.04 (0.10) | -0.16 to 0.23 | 0.09 (0.10) | -0.10 to 0.29 | 0.12 (0.08) | -0.04 to 0.29 |
| Fat limits and bans | 0.16 (0.07) | 0.01 to 0.30 | 0.12 (0.07) | -0.02 to 0.26 | 0.14 (0.08) | -0.01 to 0.30 | 0.11 (0.09) | -0.07 to 0.28 |
| Child marketing restrictions | -0.25 (0.11) | -0.47 to -0.03 | -0.24 (0.12) | -0.48 to -0.01 | -0.29 (0.11) | -0.50 to -0.07 | -0.24 (0.12) | -0.48 to 0.00 |
| Breast milk code | 0.14 (0.10) | -0.07 to 0.34 | 0.09 (0.10) | -0.10 to 0.28 | 0.12 (0.11) | -0.09 to 0.34 | 0.14 (0.12) | -0.10 to 0.37 |

1. **Full implementation**

|  | **Original model** | | **With other imp control** | | **With control for total FTAs** | | **With control for US BITs, US FTAs, and Total FTAs** | |
| --- | --- | --- | --- | --- | --- | --- | --- | --- |
|  | **Beta (SE)** | **95% CI** | **Beta (SE)** | **95% CI** | **Beta (SE)** | **95% CI** | **Beta (SE)** | **95% CI** |
| Tobacco taxes | -0.05 (0.10) | -0.25 to 0.14 | -0.07 (0.09) | -0.25 to 0.12 | -0.10 (0.10) | -0.29 to 0.09 | -0.10 (0.11) | -0.31 to 0.11 |
| Smoke free places | 0.14 (0.13) | -0.11 to 0.39 | 0.27 (0.13) | 0.01 to 0.52 | 0.20 (0.13) | -0.05 to 0.45 | 0.29 (0.15) | 0.01 to 0.58 |
| Graphic warnings | -0.28 (0.09) | -0.45 to -0.10 | -0.31 (0.07) | -0.45 to -0.16 | -0.26 (0.09) | -0.44 to -0.08 | -0.30 (0.11) | -0.53 to -0.08 |
| Tobacco ad bans | -0.04 (0.12) | -0.27 to 0.18 | -0.11 (0.10) | -0.30 to 0.08 | -0.02 (0.11) | -0.23 to 0.19 | 0.06 (0.12) | -0.17 to 0.29 |
| Alcohol ad & sales restrictions | -0.30 (0.19) | -0.66 to 0.06 | 0.02 (0.08) | -0.15 to 0.18 | 0.01 (0.17) | -0.32 to 0.34 | -0.09 (0.10) | -0.29 to 0.10 |
| Alcohol taxes | 0.20 (0.10) | -0.01 to 0.40 | 0.23 (0.10) | 0.03 to 0.44 | 0.20 (0.11) | -0.02 to 0.43 | 0.20 (0.11) | -0.01 to 0.42 |
| Salt reduction | 0.14 (0.09) | -0.03 to 0.30 | 0.10 (0.10) | -0.09 to 0.30 | 0.12 (0.11) | -0.08 to 0.33 | 0.17 (0.08) | 0.00 to 0.33 |
| Fat limits and bans | 0.11 (0.09) | -0.06 to 0.28 | 0.04 (0.09) | -0.13 to 0.21 | 0.06 (0.08) | -0.10 to 0.21 | 0.04 (0.10) | -0.15 to 0.23 |
| Child marketing restrictions | -0.25 (0.11) | -0.47 to -0.03 | -0.35 (0.12) | -0.58 to -0.12 | -0.37 (0.11) | -0.59 to -0.16 | -0.27 (0.13) | -0.51 to -0.02 |
| Breast milk code | 0.09 (0.11) | -0.13 to 0.30 | 0.07 (0.11) | -0.15 to 0.30 | 0.07 (0.11) | -0.14 to 0.29 | 0.07 (0.13) | -0.20 to 0.33 |

Notes: shading shows statistically significant results.

## Bibliography

1. Craig P, Cooper C, Gunnell D, Haw S, Lawson K, Macintyre S, et al. Using natural experiments to evaluate population health interventions: new Medical Research Council guidance. J Epidemiol Community Health (1978). 2012;66: 1182–1186. doi:10.1136/jech-2011-200375

2. Ho DE, Imai K, King G, Stuart EA. Matching as nonparametric preprocessing for reducing model dependence in parametric causal inference. Political analysis. 2007;15: 199–236.

3. Stuart EA. Matching methods for causal inference: A review and a look forward. Stat Sci. 2010;25: 1–21. doi:10.1214/09-STS313

4. King G, Nielsen R. Why propensity scores should not be used for matching. Political Analysis. 2019;27: 435–454.

5. King G, Zeng L. The dangers of extreme counterfactuals. Political Analysis. 2006;14: 131–159. doi:10.1093/pan/mpj004

6. Stuart EA, Green KM. Using full matching to estimate causal effects in nonexperimental studies: examining the relationship between adolescent marijuana use and adult outcomes. Dev Psychol. 2008;44: 395–406. doi:10.1037/0012-1649.44.2.395

7. Barlow P, Allen L. The impact of trade and investment agreements on the implementation non-communicable disease policies, 2014-2019: protocol for a statistical study. medRxiv [preprint]. 2022; 2022.05.13.22274669. doi:10.1101/2022.05.13.22274669

8. Egger H, Egger P, Greenaway D. The trade structure effects of endogenous regional trade agreements. J Int Econ. 2008;74: 278–298.

9. Baier SL, Bergstrand JH. Estimating the effects of free trade agreements on international trade flows using matching econometrics. J Int Econ. 2009;77: 63–76. doi:10.1016/j.jinteco.2008.09.006

10. Dür A, Baccini L, Elsig M. The design of international trade agreements: Introducing a new dataset. The Review of International Organizations. 2014;9: 353–375. doi:10.1007/s11558-013-9179-8

11. Milner H V, Kubota K. Why the Move to Free Trade? Democracy and Trade Policy in the Developing Countries. Int Organ. 2005/02/15. 2005;59: 107–143.

12. Allen LN, Nicholson BD, Yeung BYT, Goiana-da-Silva F. Implementation of non-communicable disease policies: a geopolitical analysis of 151 countries. Lancet Glob Health. 2020;8: e50–e58. doi:10.1016/S2214-109X(19)30446-2

13. Furtado KS, Budd EL, Ying X, deRuyter AJ, Armstrong RL, Pettman TL, et al. Exploring political influences on evidence-based non-communicable disease prevention across four countries. Health Educ Res. 2018;33: 89–103. doi:10.1093/her/cyy005

14. Thow AM, Abdool Karim S, Mukanu MM, Ahaibwe G, Wanjohi M, Gaogane L, et al. The political economy of sugar-sweetened beverage taxation: an analysis from seven countries in sub-Saharan Africa. Glob Health Action. 2021;14: 1909267.

15. Barlow P, Labonte R, McKee M, Stuckler D. Trade challenges at the World Trade Organization to national noncommunicable disease prevention policies: A thematic document analysis of trade and health policy space. PLoS Med. 2018;15: e1002590.

16. Dobbin F, Simmons B, Garrett G. The global diffusion of public policies: Social construction, coercion, competition, or learning? Annu Rev Sociol. 2007;33: 449–472.

17. Valente TW, Pitts S, Wipfli H, Vega Yon GG. Network influences on policy implementation: Evidence from a global health treaty. Soc Sci Med. 2019;222: 188–197. doi:https://doi.org/10.1016/j.socscimed.2019.01.008

18. Busch ML. Overlapping Institutions, Forum Shopping, and Dispute Settlement in International Trade. Int Organ. 2007;61: 735–761.
