# Supplementary material for: US and EU Free Trade Agreements and implementation of policies to control tobacco, alcohol, and unhealthy food and drinks: A quasi-experimental analysis
Source: PLoS Med. 2023 Jan 5;20(1):e1004147. doi: 10.1371/journal.pmed.1004147 (PMC9815641; doi:10.1371/journal.pmed.1004147)
Supplement: Attachment — Submitted filename: plos_med_rr_v2.docx [file pmed.1004147.s003.docx]

19^th^ October 2022

Dear Editor(s),

Thank you for the opportunity to revise and resubmit our paper in response to the editor and reviewers’ constructive comments, suggestions, and feedback.

In the revised version of the paper we have addressed all their comments, including additional discussion of the potential reasons for our heterogeneous findings, clarifying certain methodological details, and providing additional summaries of our effect estimates. We have also fully reviewed the paper and made a series of changes throughout to add clarity.

Below we outline in further detail how we have addressed the reviewers’ comments. We have also attached a revised version of the paper with tracked changes and comment boxes to denote where each of the reviewers’ points are addressed.

Thank you for your consideration.

**Response to Reviewers**

**Editor’s comments**

1. My main comment was that out of 10 policies and 4 contexts (40 different tests), only 7 were significant in the direction that the paper focuses on (one significant in opposite direction, all others null). Whilst the authors note in the discussion that "a need to investigate why impacts may vary across regulatory domains and country-partners", I'd like to see a bit more evidence/theory-informed speculation here. Why might it be that FTAs impeded child food marketing restrictions, but not tobacco ad bans or alcohol ad and sales restrictions (ie why does the impact on essentially the same policies differ between commodity)? Similarly, why might FTAs impede smoke free places, but not tobacco ad bans/taxes (ie why do they impact on some but not all tobacco control efforts)? I suspect this is something to do with both how different commodities are 'valued' in society (ie tobacco universally considered 'bad', alcohol less so); and the co-effects of different policies (ie taxes are revenue raising, not JUST health promoting).

To address the editor’s comment, we have now expanded the section of the Discussion where we speculate on the potential reasons for differences in our effect estimates across regulatory domains and commodities. Please see p.16-17 we have added this text. In particular, we note that variation in the relationship between FTA participation and the implementation of similar policies for different commodities could be explained by a wide range of factors, such as differences in the degree of contentiousness of a particular policies targeting different commodities, and the novelty of the policy and existing implementation levels prior to our study period. We have also added a section to the Discussion speculating on the potential reasons for variation in the association between FTA participation and the implementation of different policies within the same commodity category. This may include, for example, differences in the ability of industry actors to craft arguments that relate different policies to FTA rules, and differences in the visibility of economic benefits of the policies in addition to health benefits.

***

**Requests from the editors**

1. Please structure your abstract using the PLOS Medicine headings (Background, Methods and Findings, Conclusions).

Now amended (see p.2-3)

2. Please avoid assertions of primacy (“We conducted the first…”) in your Abstract Background. Instead, the final sentence should clearly state the study question.

We have now removed assertions of primacy in the Abstract and updated the last line of the Background to state the question assessed in the study (see p.2).

3. Assertions of primacy are also present in the Discussion and should be tempered accordingly (e.g., “to our knowledge”, or similar).

We have now removed these from the Discussion and stated instead that our study adds ‘new insight into the statistical relationship’ we assess, rather than the first insight (see eg p.15)

4. In the Abstract Methods and Findings, please include the study design and main outcome measures.

Now added (see p.2-3).

5. In the last sentence of the Abstract Methods and Findings section, please describe the main limitation(s) of the study's methodology.

Now added (see p.2-3)

6. Please remove the Funding section from your Abstract.

Now removed.

7. Please include continuous line numbering throughout your manuscript to facilitate further review.

Now added.

8. At this stage, we ask that you include a short, non-technical Author Summary of your research to make findings accessible to a wide audience that includes both scientists and non-scientists. The Author Summary should immediately follow the Abstract in your revised manuscript. This text is subject to editorial change and should be distinct from the scientific abstract. Please see our author guidelines for more information: <https://journals.plos.org/plosmedicine/s/revising-your-manuscript#loc-author-summary>

Now added (see p.4-5)

9. Please place citations within square brackets and preceding punctuation (e.g., [1]). This also applies to your appendices.

Now amended.

10. Please organise and cite your appendices as outlined here: <https://journals.plos.org/plosmedicine/s/supporting-information>

Now amended.

11. The footnotes in Appendix 4 appear to be labelled incorrectly.

Now corrected, thank you.

12. Please ensure that the study is reported according to the STROBE guideline, and include the completed STROBE checklist as Supporting Information. Please add the following statement, or similar, to the Methods: "This study is reported as per the Strengthening the Reporting of Observational Studies in Epidemiology (STROBE) guideline (S1 Checklist)." The STROBE guideline can be found here: <http://www.equator-network.org/reporting-guidelines/strobe/>

Now added (see p.11).

13. When completing the checklist, please use section and paragraph numbers, rather than page numbers.

Now amended.

14. Table 1: Please define Free Trade Agreement (FTA) in the legend.

Now amended (see p.27).

15. Figure 4: Please define the 95% confidence interval bars in the legend.

Now amended (see p.32).

16. Reference 26 (your protocol) is a preprint: please add [preprint] to the reference to identify it as such. Further details can be found here: <https://journals.plos.org/plosmedicine/s/submission-guidelines#loc-references>

Now amended.

***

**Reviewer 1’s comments**

1. Thanks for the opportunity to review your manuscript. My role is as a statistical reviewer, so my review concentrates on the study design, data, and analysis that are presented. I have put general questions first, followed by queries relevant to a specific section of the manuscript (with a page/paragraph reference).

   This manuscript examines whether participation in a free-trade agreement is associated with the implementation of policies that target tobacco/alcohol/unhealthy food and drinks. Data is at the country level from three time points (2014, 2016, 2019), policy implementation data comes from WHO country surveys (based on WHO recommended policies) and information on free trade agreements is from collaborative database. A range of additional covariates is available for secondary analysis of heterogeneity of the TIA effect. The WHO specific recommended policies includes tobacco and alcohol taxes and restrictions, graphic warnings on tobacco packing, legislation on breast-milk substitutes and policies that target sodium, sat and trans fats, and food marketing to children. In the main analyses, the outcomes are assessed as unimplemented or fully implemented (and with alternative coding considered in sensitivity analyses). The 'exposure' variable for the main analysis is binary (part of TIA/not part of TIA). In the main analysis a matching process (based on Manholobis distance of the matching variables) to limit confounding is used. Where balance wasn't achieved, the covariate was included in the main logistic regression model. The protocol is very detailed (and well explained), there are some deviations from this (detailed in app 5), these are quite reasonable and reflect changes required once features of the final dataset were apparent (e.g. not enough variation to get sufficient sub-groups for some secondary analyses). Limitations of the study design are well articulated in the discussion.

Thank you. We are grateful to the reviewer for the time and effort that they have dedicated to providing valuable feedback on our manuscript.

2. How is Mahalanobis distance calculated for differences in the region each country belongs to? I am more familiar with Mahalanobis distance calculated for continuous variables and I wasn't sure how it was done here.

To perform matching on the region variable we transformed this categorical variable ‘region’ into a series of binary region dummies taking the values of 1 for belonging to a particular region or 0 otherwise, and then calculated the distance measures using the standard procedure for numerical variables.

We have now clarified this on p.10 where we describe the variables in our models.

3. With the Mahalanobis matching approach, is there a straightforward way to assess common support, i.e. that there is sufficient overlap between the FTA groups in the covariates or are the countries with FTAs fundamentally different from those without?

For Mahalanobis distance matching, there is not a test analogous to the test of common support for propensity scores whereupon the distribution of propensity scores in treatment and control groups is compared. Instead, the main test akin for assessing the quality of matching on the Mahalanobis distance is to assess the overlap in the values and distribution of covariates for countries with and without the ‘treatment’ – in this case US or EU FTAs.^1^ We have included these covariate balance checks in the paper in Figure 3, which compares absolute standardized differences in the means of each covariate before and after matching. We further assessed a range of additional measures of covariate balance (e.g. to assess overlap in the variance of each covariate), as reported in S1 Appendix and S3 Table.

We have now clarified this on p.9. Here we also refer the reader to the Appendix where we describe the covariance balance tests in more detail.

4. My usual area of expertise is far away from free-trade agreements - so the following question is probably more for my own understanding than any issue with the manuscript. Are the FTAs similar enough to be that we can represent them as a universal, binary covariate? I ask because it seems that mainstream media reporting around FTAs always seems to emphasise differences between FTAs (for my country at least the reporting is always that 'we were ripped off compared to country XX') and I am curious if perhaps FTAs are broadly similar and I have a distorted impression of reality.

The reviewer is indeed correct that there are certain differences in FTAs, but many FTAs with a particular country (e.g. US) also exhibit strong overlap with other deals with that country, as FTA clauses are often ‘copy-pasted’ from one agreement to the next.^2^

To clarify this point we have now added a sentence to the ‘Introduction’ noting that despite certain differences, FTAs with the US and EU agreements have common components. Please see p.2 where we have made this change.

However, we appreciate the point of nuance highlighted by the reviewer, and have added a point to the Discussion noting that whilst FTAs are markedly similar to one another, they are not 100% identical. It is therefore important to bear this caveat in mind when considering the applicability of our findings to a specific country’s FTA arrangements. Please see p.17 where we have added this point.

5. P6, Paragraph 2. What criteria were used to decide between purely regression adjustment and matching (once it was clear first order models weren't appropriate)?

We performed matching instead of regression adjustment because the specific counterfactual or ‘control group’ (i.e. those countries not participating in US or EU FTAs), and their comparability with the ‘treatment group’ (i.e. those countries participating in US or EU FTAs), is not transparent with regression. This can lead to treatment effects being estimated based on unknown comparisons, or yield causal estimates based on implausible counterfactual comparisons. By contrast, matching permits transparent comparison of the treatment and control groups, and can lead to more valid causal estimates by constructing a comparison group that is more similar to the treatment group.

We have now clarified this on p.8-9 where we describe our study methodology.

6. P7, Paragraph 3. This could be my reading of it but it seems like the part: "FTAit is one of two FTA indicators in country i with coefficient B1: i) US FTA participation, or ii) EU FTA participation, lagged by 1 year (t-1) to allow for a delayed effect; we estimate separate models for US FTAs and EU FTAs." is contradictory, if there is an indicator variable for EU or US participation woudn't that make the variable unvarying if separate models for US/EU are run?

Thank you for highlighting this point of confusion. To clarify, we have two separate variables, which are dichotomous indicators of participating in a particular FTA. One of these indicators, ‘USFTA_it-1_’ captures whether country i participated in a US FTA (1) or not (0) in year t-1. The second indicator, ‘EUFTA_it-1_’ captures whether country i participated in an EU FTA (1) or not (0) in year t-1. We estimated separate models for each FTA type. To clarify this we have now written out the two separate models, as follows:

Equation 1. Implementation_it_ = B_0_ + B_1_USFTA_it-1_ + B_2_X_it-1_ + B_3_Wave_t_ + e_it_

Equation 2. Implementation_it_ = B_0_ + B_1_EUFTA_it-1_ + B_2_X_it-1_ + B_3_Wave_t_ + e_it_

Please see p.10 where we have made this change and clarified the construction of these variables.

7. With this model each country will contribute multiple 'rows' of data regression model, i.e. there are repeated measurements (of different times) of the same countries. Is this correlation accounted for by the block bootstrap procedure?

The block bootstrap is valid for estimating standard errors (and associated confidence intervals) in the same circumstances cluster robust standard errors, including, as noted by the reviewer, where each country has repeated measurements.^3^ The block bootstrap standard error is also known to approximate the cluster-robust SE.^4^ We have now noted this and added further references on p.11.

8. Also - is this the 'simple' version of block/cluster bootstrapping where an entire block/country is selected in the sampling, or is this the more complex procedure where there is second level of sampling of years within the selected blocks? Also to confirm, is country the 'block' for the block bootstrapping?

The block bootstrap procedure we followed involved sampling matched strata from the matched sample, where each strata ID contains at least 1 country with a US or EU FTA and at least 1 country without a US or EU FTA. Since our initial analyses perform matching for each country in each year, the blocks that are selected in the sampling are the strata IDs for each country in a specific year. Thus there is no further sampling of years within these selected blocks. We have clarified this on p.11.

9. P19. Table 1. I would consider removing the p-values here, the rest of the table provides everything necessary to understand the differences.

We believe that the presentation of p-values is helpful here to enable rapid interpretation of the significance of the comparisons we present in Table 1. We believe this may be particularly helpful for readers – including legal and policy experts – who are less familiar with statistical analyses and/or are more accustomed to the presentation of p-values rather than using differences and standard errors to infer statistical significance.

10. Should the title of the table include "EU FTAs"

Yes, thank you. We have now corrected this (see p.26).

11. Under the EU FTAs there is a typo - "differesnce" should be difference.

Thank you – now corrected (see p.26).

12. P18. What was the minimum number per category you considered to be appropriate allow for sufficient statistical power?

In order to estimate our models, we required variation in the outcome variable across countries with and without either US FTAs or EU FTAs. This was not the case for sales restrictions when considered as a stand-alone variable and thus we collapsed it with advertising restrictions for our analysis. We have now clarified this on p25.

***

**Reviewer 2’s comments**

1. This is an excellent and informative paper. Well done. Before publishing there are some exceedingly minor changes to make.

Thank you. We are grateful to the reviewer for the time and effort that they have dedicated to providing valuable feedback on our manuscript.

2. p. 5 'first quant study' is a bold claim that needs nuance. A google search shows there have been lots of (non-peer reviewed?) 'studies', impact assessments and so forth in this space. Also next section suggests a few of these studies. Suggest paring back this claim or adding nuance.

We have revised the wording to present a greater degree of nuance, as follows: previous studies assessing the impacts of FTAs on NCD policies adopted qualitative methodologies and ex ante impact assessments. These studies provide important insights into potential impacts and mechanisms. In our study we add to this literature performing a quantitative study of potential impacts to assess the statistical relationship between US and EU FTA participation and the probability of regulating unhealthy commodities. This quantifies the relationship via ex post assessment of potential impacts, whilst reducing covariate confounding to improve the validity of our causal estimates.

We have now clarified the above points on p.7-8. We have also removed all references to the primacy of our study throughout the paper.

3. Typo p. 10 'All results are consistent in sign for all models'. 'Significance?'

The section of text referred to by the reviewer pertains to the consistent direction of effects observed in our robustness checks. Not all of these were statistically significant, as may be expected give the large number of covariate controls in some models. We have now clarified this on p.14.

4. P. 12 'window of opportunity' is vague. What is the window and why has this come about?

We have now modified the terminology in the section of the paper by referring to a ‘potential opportunity’. We further note that this opportunity may arise for countries currently negotiating FTAs as there is still scope to change the content of these agreements whilst they are still being drafted. We have also provided examples of deals currently under negotiation to clarify the relevance of our findings and associated opportunities for careful FTA drafting to mitigate potential effects we identify.

Please see p.17 where we have made this change.

**Reviewer 3’s comments**

1. This paper uses natural experiment methods to assess the association of US or EU Free Trade Agreement participation with the implementation of various health policies. The paper thoroughly assembles what data is available for countries as the unit of analysis, and uses a matching procedure to try and approximate an unconfounded comparison. Unfortunately, not many matched country-pairs make it through to the final analytical sample - a necessary cost of trying to remove confounding, and increase internal validity.

There are 48 'tests' of whether FTA participation effects health policies: 12 policies, by 4 combinations of EU/US FTA and Full or partial/Full classifications. Figure 4 shows these 48 'tests' as a forest plot, correctly portraying the mixed findings and lack of an overwhelming pattern. That said, there is a pattern - 9 of the 48 'tests' (19%) have 95% CI excluding the null AND a finding of worse health policy implementation if the country is a participant of a FTA, whereas only 1 'test' shows better health policy. Averaged across all 48 tests, the average percentage difference was -4.4%.

The study is clearly worthy of publication. And it will generate substantial discussion and debate - rightly so. This is the first paper to quantitatively test this hypothesis in a comprehensive manner - testing a hypothesis long suspected. I have only a few recommendations for the authors in revision.

Thank you. We are grateful to the reviewer for the time and effort that they have dedicated to providing valuable feedback on our manuscript.

2. It is not clear on reading the abstract that 48 tests were run, than many were null, etc. An indication of the PATTERN of 19% of tests showing a statistically significant decreases, compared to only one (2%) an increase, would help. Moreover, as a more thorough sense of the whole pattern, giving the average effect (-4.4%) with a 95% CI would be useful (which may require some careful math to allow for correlations of the partial / partial or full classification).

Thank you for this helpful suggestion. We have now incorporated this point into our abstract where we note that 19% (9/48) of tests showed a statistically significant decrease in the predicted probability of at least partial unhealthy commodity policy implementation, whilst 2% (1/48) showed an increase. We have further calculated a combined effect estimate for US FTAs and EU FTAs. Both estimates are calculated as an equally weighted average of effect estimates with a pooled average standard error to calculate CI. Thus, we further note in the Abstract that, on average, US FTA participation associated with a 5 percentage point (%) (95% CI: -0.17 to 0.07) decline in the predicted probability of at least partially implemented the unhealthy commodity policy in question, and EU FTA participation associated with a 5% (95% CI: -0.11 to 0.01) decline in the predicted probability of at least partially implemented the unhealthy commodity policy in question.

We have also added this to the Abstract on p.2-3, Results section on p.11-12, and Discussion section on p.14-15.

3. Perhaps point out in the paper that pulling out the 9 'positive' tests for profiling is a bit risky. To many this falls foul of a statistical testing approach. Hence my suggestions above to give the average effect size. Or alternatively, the percentage of 'tests' with the effect size stronger than -20%, between --20% to -10%, etc - to convey a focus on magnitude of the associations.

We agree and have now amended the Abstract to address the reviewer’s suggestion by presenting the average effect size and general patterning of the results. Please see our response to the reviewer’s comment no.3 above where we describe the changes we have made in this respect.

4. The matching in Table 1 does not look as good as the text of the paper implies, or the absolute standardized means in Fig 3. For example, the absolute standardized mean differences in GDP in Fig 3 nicely moves almost to nil after matching - but not in Table 1. (Actually in Table 1, the differences in GDP remain, just flipped in sign.) Either I have misunderstood Table 1, or there is perhaps an error in the data in Table 1?

Table 1 presents the comparison of countries according FTA status before we conducted matching. There are indeed large, statistically significant, differences between the characteristics of countries with and without US FTAs and EU FTAs before matching is performed. Figure 3, by contrast, shows how the (standardized) differences are then reduced after matching. Table 1 therefore presents the precise differences in covariate characteristics that our matching subsequently reduces.

To make this clearer and avoid any confusion we have made two changes. First, we have amended the text on p.11 to clarify that Table 1 portrays the large differences in covariates before matching. Second, we have modified the title of Table 1 (see p.26) to further clarify that the contents of the table pertain to covariate differences before matching, rather than after matching.

5. Box 2's title implies this is a WHO categorization - but it is actually the categorization used for analysis in this paper. Perhaps make this clearer.

Thank you for highlighting this ambiguity. We have now clarified this point by removing reference to WHO in the title and noting in the notes that the policies are recommended by WHO and categorized by the research team. See p.25 where we have made this change.

6. Congratulations to the authors on an important paper.

Thank you.

**Reviewer 4’s comments**

1. This paper shines a light on an important issue and methodologically speaking, is executed superbly. The authors write in an accessible and clear way about a highly complex and technical public health issue.

Thank you. We are grateful to the reviewer for the time and effort that they have dedicated to providing valuable feedback on our manuscript.

2. My one, very minor, comment is that the authors might consider including the UK-CPTPP deal as an important US led agreement under negotiation ( bottom of pg. 4 )

Thank you for this helpful suggestion. We have now incorporated mention of the UK-CPTPP on p.7 and p.17 where we mention US deals currently under negotiation. Specifically, we note that our findings may be relevant for the UK as it negotiates accession to this US-led agreement.
